# Effect of Vitamin D Supplementation on (25(OH)D) Status in Children 12–30 Months of Age: A Randomized Clinical Trial

**DOI:** 10.3390/nu15122756

**Published:** 2023-06-15

**Authors:** Mario Flores-Aldana, Marta Rivera-Pasquel, Armando García-Guerra, Jesús Giovanni Pérez-Cortés, Juan E. Bárcena-Echegollén

**Affiliations:** 1 Centro de Investigación en Nutrición y Salud, Instituto Nacional de Salud Pública, Avenida Universidad 655, Colonia Santa María Ahuacatitlán, Cuernavaca 62100, Mexico; mrivera@insp.mx (M.R.-P.); garciaf@insp.mx (A.G.-G.); jernestobe@gmail.com (J.E.B.-E.); 2 Instituto de Investigación en Nutrición y Salud Alimentaria, S.C. Comonfort No. 7, Colonia Centro, Cuernavaca 62000, Mexico; jesus_giovanni@hotmail.com

**Keywords:** vitamin D, cholecalciferol, ergocalciferol, nutritional deficiencies, supplements, children

## Abstract

Vitamin D (VD) deficiency (serum 25(OH)D < 50 nmol/L) affects 27.3% of preschool children in Mexico. The purpose of this study was to assess the effect of vitamin D supplementation at different doses on serum 25(OH)D concentrations in preschool children. In a randomized control trial, 222 children 12–30 months old were randomly assigned to one of four treatment groups: (1) Vitamin D2 (Ergocalciferol) 400 IU/day (*n* = 56); (2) Vitamin D2 (Ergocalciferol) 800 IU/day (*n* = 55); (3) Vitamin D3 (Cholecalciferol) 1000 IU/day (*n* = 56); or (4) multiple micronutrients (MM) non-VD (*n* = 55). Supplements were given five days/wk for three months. Serum 25(OH)D was measured at baseline and after three months. At baseline, mean serum 25(OH)D was 58.9 ± 12.6 nmol/L and 23.4% were VD-deficient. There was a statistically significant increase in serum concentrations of 25(OH)D (range across groups: +8.2 to +17.3 nmol/L). Additionally, the prevalence of vitamin D deficiency decreased after three months: for D2 400 IU, −9.0%; for D2 800 IU, −11.0%; for D3 1000 IU, −18.0%; and for MM non-VD, −2.8% (*p* < 0.05). No adverse effects were observed. VD supplementation for three months was effective for increasing serum 25(OH)D concentrations and for reducing VD deficiency in preschool children. The highest efficacy was observed by giving 1000 IU D3/d.

## 1. Introduction

Vitamin D (VD) is an essential nutrient in humans, which can be synthesized in the skin after exposure to UVB sunlight rays [1]. It can also be obtained from the intake of foods of vegetable origin (Vitamin D2: Ergocalciferol) or products of animal origin (Vitamin D3: Cholecalciferol). Fruits and vegetables are very low in VD. Animal products that naturally contain VD are salmon, eggs, and oils from fish, including cod liver oil [2]. In Mexican preschool-age children, the mean VD intake from foods was 135 IU/day. Milk, dairy, and milk-based beverages and foods contributed to 90% of their vitamin D intake [3]. However, occasional sun exposure is the main source of circulating VD is for Vitamin D and 25-Hydroxyvitamin-D 25(OH)D [2]. The main functions of VD include the maintenance of adequate serum levels of calcium and phosphorous, bone health, modulation of the immune response, and regulation of cell growth and proliferation, among others [4].

In addition to its role in bone health, VD is of importance for many metabolic and physiological processes. Conversely, VD deficiency has been associated with unfavorable outcomes for human health, among which are autoimmune and allergic diseases such as type 1 diabetes, asthma, respiratory tract infections, chronic diseases such as several types of cancer, and cardiovascular disorders [4,5,6].

It is estimated that VD deficiency (<50 nmol/L of 25(OH)D) is a global problem affecting approximately one billion people. Some possible factors associated with VD deficiency are less sun exposure and outdoor physical activity, increased BMI, and use of sunscreen [7]. In a recent nationwide survey in Mexico, a mean serum concentration of 60.93 nmol/L of 25(OH)D and a prevalence of 27.3% of VD deficiency were documented in children from one to five years of age. It was also found that less than 3% of children regularly consume a VD supplement [8].

A number of studies have assessed the effects of VD supplementation. In Italy, a study in children ages 2–15 years with moderate VD deficiency showed that a 1500 IU dose of vitamin D3/day for six months was appropriate for children to maintain normal to near-normal 25(OH)D levels [9]. Other studies have shown that vitamin D3 supplementation of 400 IU/day, 1000 IU/day, 2000 IU/day, and 4000 IU/day in children ages 9–13 years was safe and effective in raising mean concentrations of 25(OH)D in a 12-week period. They also showed that serum 25(OH)D increased in a dose-dependent manner, and higher doses resulted in higher long-term concentrations [10,11,12,13]. In a recent study carried out in overweight and obese children and adolescents ages 6–16 years, the increase in 25(OH)D levels was higher among children and adolescents supplemented with 1000 IU and 2000 IU of vitamin D3 compared with the group who received 600 IU in a period of six months. Nevertheless, children with higher BMI did not achieve serum 25(OH)D levels ≥ 50 nmol/L compared with those who achieved serum 25(OH)D > 50 nmol/L [14].

Routine, widespread VD supplementation in children has not been implemented in Mexico. Therefore, the objective of the present study was to evaluate the efficacy of supplementation with 400 IU or 800 IU of vitamin D2 and 1000 IU of vitamin D3 for three months on serum 25(OH)D levels and the prevalence of VD deficiency in children from 12 to 30 months of age.

## 2. Materials and Methods

### 2.1. Study Design and Intervention

A randomized, controlled trial with four treatment groups (i.e., factorial design) was conducted as follows: (1) Vitamin D2 (Ergocalciferol) 400 IU/day (*n* = 56); (2) Vitamin D2 (Ergocalciferol) 800 IU/day (*n* = 55); (3) Vitamin D3 (Cholecalciferol) 1000 IU/day (*n* = 56); and (4) MM non-VD (as comparison group) (*n* = 55). Supplements were given daily (from Monday to Friday) for three months. The study was conducted from September 2016 to February 2017 in daycare centers in Cuernavaca, Morelos, Mexico (18.9° N, 99.2° W, and 1510 mts above sea level).

The study was carried out according to the guidelines of the Declaration of Helsinki and was approved by the Committees on Ethics, Biosafety, and Research at the National Institute of Public Health (INSP, Cuernavaca, Morelos, Mexico). All supplements were provided free of charge. Parents/caregivers signed an informed consent to participate in the study. The study was registered with Clinical Trials, NCT03544671.

### 2.2. Setting and Participants

Eligible toddlers were healthy preschool-age children 12–30 months old of both sexes. Children who were taking a VD supplement and those who were clinically ill were excluded from the study. Children were recruited at eight public daycare centers in Cuernavaca, Mexico, affiliated with Secretaría de Desarrollo Social (SEDESOL).

Once the principal of a daycare center agreed to participate, he or she made appointments with parents/caregivers at each daycare center. At these meetings, the project coordinator and a researcher gave the parents/caregivers a detailed explanation of the objective, methods, and risks posed by the study.

### 2.3. Supplements

**Figure 1:**
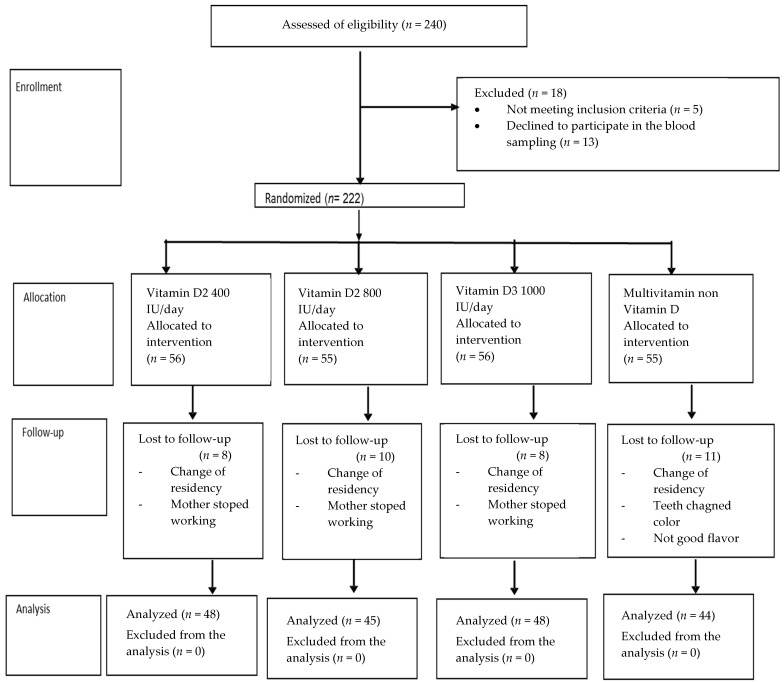
Study flow-chart of participants showing the process of children selection and enrollment, allocation to the study groups.

Children assigned to the vitamin D2 treatment groups were given Vi-dea-C^®^ (DEGORT’S Laboratory, Mexico City, Mexico), which contained 400 IU/mL. Children in the 400 IU/day received 1 mL/d, and children in the 800 IU/day received 2 mL/d. For the 1000 IU/day D3 group, D-drops™ (Woodbridge, ON, Canada) was used, and children were given one drop/d. Children assigned to the MM non-vitamin D group received 1 mL/d of Fortimin^®^, which is the standard vitamin supplement for preschool children provided by the Ministry of Health (Supplemental Table S1). Trained personnel gave supplements daily, from Monday to Friday, directly to the child according to treatment allocation and registered its consumption. Supplements were registered trademarks. Enrollment, randomization scheme, and final sample distribution by treatment group are presented according to the CONSORT diagram (Figure 1).

### 2.4. Intervention

Each toddler was randomly assigned using the Moses–Oakford method [15] for allocation to each of the treatment groups. Toddlers were stratified according to age: 12–18 mo, 19–24 mo, and 25–30 mo, and sex (male/female). Adherence was assessed by supplement consumption, recorded in milliliters in a standardized manner every day at each daycare center. If the child was ill or absent from school for more than seven days, it was considered as non-compliant.

### 2.5. Follow-Up and Measurements

At baseline and after three months, parents were scheduled on a weekday to arrive at 6 a.m. in the morning at their daycare center for blood sampling. Because children were between 12 and 30 months of age, there was no fasting overnight. Venous blood samples (5 mL) were drawn from the antecubital vein at baseline and at the end of the study. Samples were obtained by a trained doctor according to protocol procedures established by the Biosafety Committee at the National Institute of Public Health (INSP). Serum and plasma samples were separated within 4 h of collection and stored at −70 °C until defrosted for analysis at the INSP nutrition laboratory. Total serum 25(OH)D concentration and parathyroid hormone (PTH) were measured using a chemiluminescent microparticle assay -CMIA- with an Architect^®^ analyzer (Abbott Diagnostics, Lake Forest, IL, USA), with an overall interassay CV of <5.5%.

Hemoglobin concentration was taken for screening procedures, at baseline and then at three months after the start of supplementation, and determined in capillary blood samples obtained by finger prick and measured in Portable Photometer (HemoCue Hb 201+ (Hemocue.Hb201, Angelholm, Sweden) [16].

### 2.6. Outcome

We evaluated the effect of VD supplementation with 400 IU and 800 IU of vitamin D2, and 1000 IU of vitamin D3 on serum 25(OH)D concentrations in preschool-age children. A 25-hydroxyvitmin D level less than 50 nmol/L was defined as VD deficiency [17].

### 2.7. Dietary Assessment

Information on diet was obtained through a semi-quantitative food frequency questionnaire (SFFQ) with 123 items referring to the previous seven days, as described elsewhere [18].

### 2.8. Compliance

Supplement consumption was calculated as the number of days the child received the supplement in grams/day or drops/day. The median consumption was calculated and compared over the analysis sample for each treatment group (Supplemental Table S2).

### 2.9. Anthropometric Measurements

Weight and height/length were collected by trained nutritionists and standardized with international procedures [19,20]. Weight was measured with an electronic scale (Tanita^®^) with a capacity of 140 kg and an accuracy of 100 g. Height/length was measured using a wooden stadiometer with a capacity of 2 m and precision of 1 mm (Short Productions, Olney, MD, USA). *Z*-scores were calculated for weight and height/length using the 2006 WHO standards. Stunting was defined as a length for age *Z*-score < −2 SD; underweight, as a weight for age *Z*-score < −2 SD; wasting, as a weight for length/height *Z*-score < −2 SD; and overweight or obesity, as body mass index (BMI) for age *Z*-score > +2 SD [21].

### 2.10. Socioeconomic Variables

Children’s ages and dates of birth were provided at baseline by the parents/caregivers. A household well-being index (HWI) was constructed using household information: type of floor, wall and ceiling materials, number of persons living in the household, and domestic appliances. Principal components analysis was used to construct the HWI [22]. Information on the mother´s educational level and access to health care was also obtained.

### 2.11. Sample Size

Sample size and power were calculated considering a baseline level of 25(OH)D between 41–60 nm/L. A sample of 250 children was estimated to achieve 80% statistical power to estimate a difference of 10 nm/L among treatment groups, assuming a loss-to-follow-up of 20% [23,24].

### 2.12. Statistical Analysis

Baseline characteristics were compared across treatment groups using ANOVA and *χ*^2^ tests. Means and standard deviations were calculated for continuous variables and percentages for categorical variables. Multiple linear regression analysis was used to estimate differences in 25(OH)D among treatment groups, adjusting for differences at baseline and clustering for daycare centers. A statistical significance level of 0.05 was used. Data management, processing, and statistical analysis were done using STATA v. 13 (StataCorp.^®^ 2013. Stata Statistical Software: Release 13. College Station, TX, USA: StataCorp LP).

## 3. Results

The number of children assessed for eligibility was 240. Two hundred and twenty-two healthy toddlers were randomized (Figure 1). Eighteen children were excluded, five did not meet exclusion criteria, and thirteen declined to participate because mothers were afraid of blood sampling. Dropout rates were between 13 and 25% across groups. The main reason for dropouts was that mothers stopped working; therefore, children did not continue in the daycare center, and change of residency.

On average, the compliance rate was 75.8% ± 15.1% considering the total days of supplement consumption (Supplemental Table S2). Supplements were well accepted by the toddlers except for the MM non-VD supplement. Mothers’ argument was that children’s teeth changed color. Only one child was considered non-compliant but completed the study.

Children’s characteristics at baseline for each treatment group are presented in Table 1. The mean age was 28.7 ± 10.7 mo. On average, children who received the 1000 IU of vitamin D3 and the MM non-VD supplement were older than children who received the vitamin D2 supplements (*p* < 0.05). There were also differences in weight for length/height *Z*-score among treatment groups (*p* < 0.05). Children in the 400 IU and MM non-VD groups had higher scores, compared to the other groups. However, no differences in *Z*-scores nor in nutritional status were observed after three months of study. At baseline, the mean VD intake was 99.7 ± 183.7 IU/day.

No differences among treatment groups were observed at baseline in relation to biochemical measures. There was a significant and positive change in serum 25-OH-D concentrations over three months of the trial among children in each of the three VD groups but not in the MM non-VD group (Table 2).

For 25-OH-D, no difference was observed between the 400 IU D2 and the 800 IU D2 groups. For the other treatment groups, pairwise comparisons were statistically significant, with the largest change in the 1000 IU D3 group, followed by the 800 IU D2 group.

**Figure 2:**
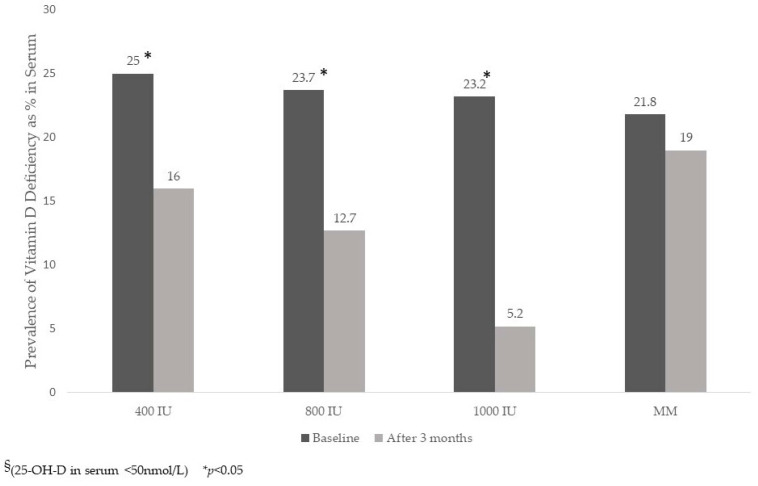
Prevalence of vitamin D deficiency ^§^ in children 12–30 months of age before and after supplementation by treatment group.

A statistically-significant decrease was observed in iPTH concentrations for the three VD-supplemented groups but not in the MM non-VD group. Pairwise comparisons were statistically-significant only when comparing each of the three VD-supplemented groups vs. the MM non-VD group. The correlation between iPTH and 25-OH-D was between −0.16 and −0.15 at baseline and after three months (*p* < 0.05). No significant changes in hemoglobin were observed. The prevalence of VD deficiency decreased in all three VD-supplemented groups, with the largest effect observed in the 1000 IU D3 group (Figure 2) 

## 4. Discussion

This randomized clinical trial shows that VD supplementation in doses of 400 IU (D2), 800 IU (D2), and 1000 IU (D3) five times per week for three months was effective in increasing serum 25(OH)D concentrations and reducing VD deficiency in children 12–30 months of age. The best efficacy was observed by giving 1000 IU D3.

There are few studies assessing the efficacy of daily doses of vitamin D2-D3 supplementation in increasing the serum 25(OH)D levels in preschool children compared with a control group [9,10,11,12,13]. VD recommendations vary across countries. Despite all efforts made, there is no agreement on the appropriate recommendations for toddlers and children [25]. As an example, in the United States, experts have recommended at least 400 IU/day for children over one year of age [26], the same as in Mexico [27]; in contrast, the recommendation in Europe is 1000 IU/day for children 1–10 years old [28]. These recommendations are intended to maintain serum 25(OH)D concentrations ≥ 25 nmol/L to increase calcium absorption from the diet, which in turn would promote bone health [25].

Given that natural dietary sources are scarce in VD and that sun exposure is not as frequent as can be expected, even in sunny places such as Mexico [3], children not receiving supplementation or vitamin D-fortified foods are at risk of VD deficiency and, consequently, of inadequate bone mineralization [26]. In our study, the dose response per IU of VD in serum 25(OH) D concentrations was 2.05 nmo/L, 1.31 nmol/L, and 1.73 nmol/L for 400 IU D2, 800 IU D2, and 1000 IU D3 doses, respectively. The results of a meta-regression analysis in 28 randomized clinical trials carried out in children under four years showed that among children between 0–12 mo of age, for each 100-IU/day, the corresponding increase in serum 25(OH)D was, on average, 1.92 nmol/L (95% CI: 0.28, 3.56), adjusted by residual heterogeneity [29]. Additionally, in children three to nine years old, random-effects meta-regression showed that each 100 IU/day increase in VD supplementation was associated with an average of 2.49 (95% CI: −0.24, 5.22) nmol/L increase in achieved 25(OH)D concentration [29]. The results of our study are within the range of the effects reported in this meta-analysis. On the other hand, the correlation between baseline and final 25-OH-D levels with PTH was in the range of −0.15 to −0.30, which has been observed in other studies [30].

The effect of VD supplementation observed in our study is in accordance with a double-blind VD supplementation trial carried out in children in the northeastern U.S. The study supplemented with three different dosages of vitamin D3 (600, 1000, and 2000 IU/day) and found that children with the highest dosage of vitamin D3 (2000 IU/day) had the highest mean serum concentration of vitamin D3 after six months of supplementation compared with the other two groups (33.1 vs. 26.3 and 27.5 ng/mL; *p* < 0.001, respectively) [31].

In another RCT conducted in the U.S., children 7 months to 10 years of age received weekly doses equivalent to 400 IU/day or 1000 IU/day during six months. An average increase of 11.8 nmol/l was observed in the 400 IU group and 19.3 nmol/L in the high dose group (similar to our findings: 8.2 nmol/L and 17.3 nmol/L, respectively) [32]

Consistently, in our study, we observed a higher increase in serum 25(OH)D levels associated with a larger vitamin D dose, which is in agreement with the two aforementioned studies.

Among the strengths of our study, we can consider its randomized design, with the inclusion of a comparison group: the MM non-vitamin D-supplemented group. Additionally, our study tested the efficacy of two forms of oral VD supplementation (namely D2 ergocalciferol and D3 cholecalciferol) at different doses ranging from 400 IU/day to 1000 IU/day (10 to 24 µg/d).

Finally, there were practical difficulties and reasons for testing two available doses of D2 vs. a higher dose of D3; not available in Mexico at the time. We tried to get the supplements from a private pharmaceutical company in Mexico City. Unfortunately, the fact that no one was manufacturing these supplements at the time, and they were not registered at the Mexican equivalent of the FDA, would involve a very time-consuming, as well as an extremely costly process. Therefore, we gave the children what was commercially available in Mexico, which makes sense, from the point of view of the practical implications of the study, and tried an imported D3 supplement, in the hope that the latter would be more effective, as it turned out to be.

In our study, the supplements were registered trademarks, and the field workers knew which supplement each child received. However, the researchers and the data analyst were blind in relation to the treatment.

## 5. Conclusions

VD supplementation in doses of 400 IU (D2), 800 IU (D2), and 1000 IU (D3) per day, five times per week for three months, was effective in increasing serum 25(OH)D concentrations and reducing VD deficiency in children 12–30 months of age. The highest efficacy was observed by giving 1000 IU/day D3. Our study adds to the evidence of the effect of VD supplementation on VD status and in the prevention of VD deficiency among toddlers and preschool children, which has long-term benefits for bone health, growth, and other important health-related outcomes at this early stage of the life cycle. More studies are needed to assess these effects in this age group.

## Figures and Tables

**Table 1:** General characteristics and Nutritional status of the children who completed the study at baseline and after three months, by treatment group.

	Baseline					After 3 Months				
	Treatment					Treatment				
	400 IU	800 IU	1000 IU	MM	*p*	400 IU	800 IU	1000 IU	MM	*p*
	*n* = 56	*n* = 55	*n* = 56	*n* = 55		*n* = 48	*n* = 45	*n* = 48	*n* = 44	
Age (month) ^a^	26.4 ± 0.9	26.5 ± 1.0	30.3 ± 1.6	33.7 ± 1.5	0.001	30.5 ± 1	30.5 ± 1.1	32.5 ± 1.7	35.9 ± 1.7	0.036
Gender										
Male (%) ^b^	56.5	56	58.2	60.8	0.954	50.9	55.7	55.7	57.9	0.913
Female (%) ^b^	43.5	44	41.8	39.2		49.1	44.3	44.3	42.1	
Nutritional status										
BMI/age *Z*-score ^a^	0.20 ± 0.1	−0.11 ± 0.11	−0.17 ± 0.12	0.06 ± 0.14	0.087	0.19 ± 0.11	−0.04 ± 0.13	−0.09 ± 0.12	−0.06 ± 0.16	0.279
Weight/Length	0.16 ± 0.09	−0.20 ± 0.11	−0.24 ± 0.12	−0.03 ± 0.14	0.046	0.16 ± 0.10	−0.11 ± 0.12	−0.15 ± 0.12	−0.06 ± 0.16	0.185
Z-scorea										
Length/age Z-score ^a^	−0.33 ± 0.18	−0.54 ± 0.21	−0.64 ± 0.17	−0.67 ± 0.22	0.727	−0.18 ± 0.2	−0.44 ± 0.24	−0.54 ± 0.18	−0.24 ± 0.25	0.583
Stunting (%) ^b^	7.6	10.9	11.3	10.4	0.89	7.4	8.8	9.1	10.8	0.956
Adequate (%) ^b^	92.4	89.1	88.7	89.6		92.6	91.2	90.9	89.2	
Dietary										
Total Kcal/day ^a^	1236 ± 66	1293 ± 73	1191 ± 60	1190 ± 55	0.444	1429.4 ± 106.7	1563.9 ± 115.1	1422.3 ± 89.1	1570.9 ± 89.4	0.419
Vit D (IU)/day ^a^	86.3 ± 20.3	92.4 ± 25.0	89.1 ± 25.2	124.1 ± 32.7	0.601	98.1 ± 24.4	104.0 ± 30.6	95.7 ± 28.3	121.7 ± 24.4	0.589
Calcium (mg/day)	756.9 ± 48.7	752.8 ± 51.0	712.3 ± 57.3	736.8 ± 55.9	0.953	877.02 ± 62.6	914.22 ± 67.0	817.9 ± 73.8	807.1 ± 74.3	0.940
Socioeconomic status										
Low	36.2 ± 5.8	32.0 ± 5.4	32.8 ± 5.8	37.3 ± 6.8	0.589					
Medium	36.2 ± 5.8	36.0 ± 5.6	47.8 ± 6.1	33.3 ± 6.6						
High	27.6 ± 5.4	32.0 ± 5.4	19.4 ± 4.9	29.4 ± 6.4						
Mother’s Educational Level										
Elementary school or less ^I^ (%)	9.1 ± 3.6	4.1 ± 2.3	12.3 ± 4.1	13.7 ± 4.8	0.444					
Middle school ^II^ (%)	30.3 ± 5.7	41.1 ± 5.8	40.0 ± 6.1	27.5 ± 6.3						
High school ^III^ (%)	40.9 ± 6.1	31.5 ± 5.5	33.8 ± 5.9	39.2 ± 6.9						
College or more ^IV^ (%)	19.7 ± 4.9	23.3 ± 5.0	13.9 ± 4.3	19.6 ± 5.6						
Access to health care										
Yes (%)	82.1 ± 4.7	86.3 ± 4.0	84.1 ± 4.6	82.3 ± 5.4	0.904					
No (%)	17.9 ± 4.7	13.7 ± 4.0	15.9 ± 4.6	17.7 ± 5.4						

^a^ Values of means ± standard errors are shown; ^b^ Proportions are presented (%); Vitamin D2 (Ergocalciferol). Cut-off points BMI/Age; Low Weight < −2 SD; Adequate ≥ −2 SD & ≤2 SD; Overweight > 2 SD & ≤3 SD; Cut-off points Weight for Length; Wasting < −2 SD; Adequate ≥ −2 SD & ≤2 SD; Overweight > 2 SD & ≤3 SD; Cut-off points Length for Age; Stunting < −2 SD; Adequate ≥ −2 SD, The cut-off points are defined by the SDs with respect to the mean of each anthropometric indicator. ^I^ No Education, elementary school (complete/incomplete), middle school (incomplete); ^II^ Middle school (complete), high school (incomplete); ^III^ High school (complete), college (incomplete); ^IV^ College (complete), post-graduate studies.

**Table 2:** Difference in biochemical indicators between baseline and after three months in children who completed the study by treatment group.

	400 IU D2	800 IU D2	1000 IU D3	MM	400 IU D2 vs. 800 IU D2	400 IU D2 vs. 1000 D3	400 IU D2 vs. MM	800 IU D2 vs. 1000 D3	800 IU D2 vs. MM	1000 IU D3 vs. MM
25(OH)D (nmol/L)	*n* = 56	*n* = 55	*n* = 56	*n* = 55						
Baseline	59.1 ± 1.63	58.7 ± 1.71	59.8 ± 1.88	57.7 ± 1.54	0.40 ±2.36	−0.7 ± 2.49	1.0 ± 2.30	−1.10 ± 2.54	1.00 ± 2.30	2.10 ± 2.43
3 months	67.9 ± 2.26	70.5 ± 2.53	79.5 ± 2.18	56.0 ± 2.24	−2.30 ± 3.38	−11.6 ± 3.14 *	11.9 ± 3.19 *	−9.0 ± 3.33 *	14.5 ± 3.38 *	23.5 ± 3.12 *
Change	8.2 ± 190	10.5 ± 2.18	17.3 ± 2.03	1.4 ± 1.73	−2.3 ± 2.88	−9.1 ± 2.78 *	6.80 ± 2.58 *	−6.80 ± 2.97 *	9.10 ± 2.79 *	15.90 ± 2.70 *
iPTH (pg/mL)	*n* = 56	*n* = 55	*n* = 56	*n* = 55						
Baseline	44.9 ± 1.83	50.2 ± 2.46	48.9 ± 1.87	47.5 ± 2.02	−5.3 ± 3.05	−4.0 ± 2.61	−2.6 ± 2.72	1.3 ± 3.08	2.7 ± 3.18	1.4 ± 2.75
3 months	35.4 ± 2.15	36.6 ± 2.09	32.5 ± 1.80	35.5 ± 2.00	−1.2 ± 3.00	2.9 ± 2.80	−1.0 ± 2.95	4.1 ± 2.74	1.1 ± 2.79	−3.0 ± 2.58
Change	−11.4 ± 2.16	−12.8 ± 2.20	−16.0 ± 2.02	−3.0 ± 1.52	1.4 ± 3.08	4.6 ± 2.95	−8.4 ± 2.68 *	3.2 ± 2.98	−9.8 ± 2.69 *	−13 ± 2.56 *
Hemoglobin (g/dL)	*n* = 56	*n* = 55	*n* = 56	*n* = 55						
Baseline	12.9 + 0.20	12.8 + 0.20	12.6 + 0.20	12.8 + 0.17	0.1 ± 0.28	0.3 ± 0.28	0.1 ± 0.26	0.2 ± 0.28	0 ± 0.26	−0.2 ± 0.26
3 months	12.8 + 0.20	13.1 + 0.10	12.4 + 0.10	13.2 + 0.20	−0.3 + 0.23	−0.3 ± 0.22	−0.4 ± 0.28	0 ± 0.14	−0.1 ± 0.22	−0.1 ± 0.21
Change	−0.2 + 0.18	0.3 + 0.20	−0.2 + 0.20	−0.4 + 0.31	−0.5 + 0.27	−0.5 + 0.26	0.2 ± 0.35	0 ± 0.28	0.7 ± 0.37	0.7 ± 0.36

* *p* < 0.05 between treatment groups.

## Data Availability

The data presented in this study are available upon request to the corresponding author.

## Supplementary Materials

The following supporting information can be downloaded at: https://www.mdpi.com/article/10.3390/nu15122756/s1, Table S1. Vitamin Composition; Table S2. Consumption Compliance Rate by Treatment Group. Table S3. Serum 25-OH-D (nmol/L) Parameters and data distribution analysis. Figure S1. Baseline Correlations. Figure S2. After 3 months correlations.
