# Supplementary material for: Effect of Vitamin D Supplementation on (25(OH)D) Status in Children 12–30 Months of Age: A Randomized Clinical Trial
Source: Nutrients. 2023 Jun 15;15(12):2756. doi: 10.3390/nu15122756 (PMC10304884; doi:10.3390/nu15122756)
Supplement: Supplementary file 1 [file nutrients-15-02756-s001.zip › nutrients-2408386-supplementary.pdf]

Table S1. Vitamin Composition.

VI_dea-C_ (D2) 400* IU	VI_dea-C_ (D2) 800**IU	VITAMIN D DROPS (D3)	FORTIMIN (MM)***
Retinol (Vitamin A Palmitate) 500,000 IU<br><br>Ergocalciferol (Vitamin D2) 40,000 IU<br><br>Ascorbic acid (Vitamin C) * 3,000 mg (1)<br><br>Sodium ascorbate 3,375 mg (1,2)<br><br>*Total ascorbic acid (vitamin C) 6,000 mg<br><br>Vehicle 100mL	Retinol (Vitamin A Palmitate) 500,000 IU<br><br>Ergocalciferol (Vitamin D2) 40,000 IU<br><br>Ascorbic acid (Vitamin C) * 3,000 mg (1)<br><br>Sodium ascorbate 3, 375 mg (1,2)<br><br>*Total ascorbic acid (vitamin C) 6,000 mg<br><br>Vehicle 100mL	Vitamina D3 (cholecalciferol) 1000 IU	Riboflavin 5-phosphate sodium 0.060 g<br><br>Thiamine hydrochloride (Vitamin B1) 0.055 g<br><br>Pyridoxine hydrochloride (Vitamin B6) 0.075 g<br><br>Cyanocobalamin (Vitamin B12) 0.055 mg<br><br>Folic acid (Vitamin B9) 3.750 mg<br><br>Ascorbic acid (Vitamin C) 3.0 g<br><br>Ferrous sulfate heptahydrate 4.978 g (equivalent to 1 g of iron)<br><br>Zinc sulfate monohydrate 2.744 g (equivalent to 1 g of Zinc)

* 1 mL = 400IU; ** 2 mL 800IU; *** 1 mL.

Table S2. Consumption Compliance rate by treatment.

TREATMENT	DAYS OF SUPPLEMENT CONSUMPTION (N)	CONSUMPTION COMPLIANCE RATE* (%)
400 D2	65.1 ± 22.9	74.6 ± 20.5
800 D2	66.6 ± 20.5	79.2 ± 13.9
1000 D3	75.9 ± 38.8	79.0 ± 10.8
MM	65.1 ± 29.4	70.5 ± 15.4

Means (%) ± standard error are shown. * Considering total days of supplement consumption.

Table S3. Serum 25-OH-D (nmol/L) Parameters and data distribution analysis.

TREATMENT	STAGE	MEAN	SD	MEDIAN	RANGE	COEF. VARIATION	FOLD INCREASE
400 IU D2	Baseline	59.1	12.19	52.3	38.1-89.9	20.62	1.15
	3-months	67.9	15.7	69.3	35.3-100.8	23.12	
800 IU D2	Baseline	58.7	12.67	57.8	35.4-93.4	21.58	1.20
	3-months	70.5	17.0	71.5	37.3-105.8	24.11	
1000 IU D3	Baseline	59.8	14.10	56.8	32.1-95.6	23.59	1.33
	3-months	79.5	15.1	77.5	45.0-117.3	18.99	
MM -NO VIT D-	Baseline	57.7	11.37	58.7	37.3-81.6	19.97	0.97
	3-months	56.0	15.0	57.7	27.6-90.3	26.78	

	vitD_b~l	pth_ba~l	vitDIU~t	energy~t	calciu~t	WHZ_ba~l	BMIZ_b~l
vitD_basel*	1.0000						
pth_basel	-0.1590	1.0000					
	0.0406**						
vitDIUtot	0.0905	-0.0447	1.0000				
	0.3076	0.6189					
energy_tot	-0.0874	0.1936	0.3544	1.0000			
	0.3245	0.0298	0.0000				
calcium_tot	-0.0631	0.0449	0.5940	0.6891	1.0000		
	0.4773	0.6172	0.0000	0.0000			
WHZ_basel	0.0191	0.0523	0.1358	-0.0141	0.1182	1.0000	
	0.8185	0.5383	0.0932	0.8620	0.1443		
BMIZ_basel	-0.0002	0.0689	0.1079	-0.0446	0.0724	0.9762	1.0000
	0.9980	0.4185	0.1871	0.5867	0.3772	0.0000	
LAZ_basel	-0.0154	-0.0394	0.0897	0.0937	0.1153	0.0792	-0.1159
	0.8534	0.6435	0.2735	0.2523	0.1585	0.2462	0.0892

	LAZ_basel
LAZ_basel	1.0000

* Abbreviations:
- vitD_basel: 25-OH-D at baseline
- pth_basel: iPTH at baseline
- vitDIU_tot: Vit D intake (IU/d)
- energy_tot: Energy intake (Kcal/d)
- calcium_tot: Calcium intake (mg/d)
- WHZ_basel: Weight-for-Length Z-score at baseline
- BMIZ_basel: BMI-Z-score at baseline
- LAZ_basel: Length-for-Age Z-score at baseline

**p-value

Figure S1. Baseline Correlations.

	vitD_f~l	pth_fi~l	vDIU_fnl	energy~l	calciu~l	WHZ_fi~l	BMIZ_f~l	LAZ_fi~l
vitD_final*	1.0000							
pth_final	-0.1098	1.0000						
	0.1315**							
vitDIU_final	0.0396	-0.1457	1.0000					
	0.6297	0.0696						
energy_final	-0.0081	0.1063	0.3544	1.0000				
	0.9215	0.1865	0.0000					
calcium_fi~l	-0.0197	-0.0631	0.5940	0.6891	1.0000			
	0.8104	0.4337	0.0000	0.0000				
WHZ_final	-0.0575	-0.1192	0.1472	0.0084	0.1056	1.0000		
	0.4621	0.1463	0.0960	0.9246	0.2335			
BMIZ_final	-0.0388	-0.1351	0.1432	-0.0254	0.0756	0.9726	1.0000	
	0.6227	0.1003	0.1069	0.7756	0.3961	0.0000		
LAZ_final	-0.0532	0.0505	-0.0154	0.1060	0.0726	0.0430	-0.1740	1.0000
	0.4944	0.5355	0.8606	0.2263	0.4078	0.5803	0.0241	

* Abbreviations:

vitD_final:	25-OH-D after 3 months
pth_final:	iPTH after 3 months
vitDIU_final:	Vit D intake (IU/d) after 3 months
energy_final:	Energy intake (Kcal/d) after 3 months
calcium_final:	Calcium intake (mg/d) after 3 months
WHZ_final:	Weight-for-Length Z-score after 3 months
BMIZ_final:	BMI-Z-score after 3 months
LAZ_final:	Length-for-Age Z-score after 3 months

**p-values

Figure S2. After 3 months correlations.
